# Handgrip strength association with weaning outcome in mechanically ventilated ICU patients: a systematic review and meta-analysis

**DOI:** 10.1186/s13054-025-05729-5

**Published:** 2025-11-07

**Authors:** Henri De Noray, Noémie C. Duclos, Alexandre Boyer, Thomas Gallice

**Affiliations:** 1 Centre Hospitalo-Universitaire de Bordeaux, pôle neurosciences cliniques, Bordeaux, France; 2 Univ. Bordeaux, INSERM, BPH, Bordeaux, U1219, F-33000 France; 3 College of Health Sciences, Univ. Bordeaux, Institut Universitaire des Sciences de la Réadaptation, Bordeaux, F-33000 France; 4 Département de Médecin Intensive - Réanimation, Centre Hospitalo-Universitaire de Bordeaux, Bordeaux, France; 5 Inserm, U1045, Université de Bordeaux, Centre de Recherche Cardio- Thoracique de Bordeaux, Bordeaux, France

**Keywords:** Intensive care units, Mechanical ventilation, Airway extubation, Handgrip strength, ICU acquired weakness, Systematic review, Meta-Analysis

## Abstract

Weaning from mechanical ventilation is a critical process in the intensive care unit (ICU), and extubation failure remains associated with poor outcomes. ICU-acquired weakness has been identified as a risk factor for delayed weaning. Maximal handgrip strength (HGS) is a simple bedside measure proposed as a surrogate marker of global muscle strength. This systematic review and meta-analysis aimed to assess the association between HGS and weaning outcomes in ICU patients. A systematic search of MEDLINE, EMBASE, CINAHL, and Cochrane Library was conducted up to December 2024. We included prospective cohort studies assessing maximal HGS prior to extubation in mechanically ventilated adults, and its association with extubation failure, spontaneous breathing trial (SBT) failure, or weaning classification (simple, difficult, or prolonged). Meta-analyses pooled mean differences in HGS between outcome groups, and diagnostic accuracy was evaluated using a hierarchical summary ROC model. Risk of bias was assessed using the QUIPS tool. Seven studies were included in the review (*n* = 707 patients), and six in the meta-analysis. No significant difference in HGS was found between extubation failure and success (mean difference: − 3.62 kg; 95% CI: − 7.92 to 0.62). However, significantly lower HGS was associated with SBT failure (–3.00 kg; 95% CI: − 4.64 to − 1.36) and non-simple weaning (–3.94 kg; 95% CI: − 5.31 to − 2.58). Pooled sensitivity and specificity of HGS were 72% and 60% respectively, in predicting either extubation failure or non-simple weaning. Negative predictive values ranged from 90% to 95%, for 10% and 20% pre-test probability. Maximal HGS does not appear to significantly differentiate extubation success from failure Given the limited number of studies and their heterogeneity, further high-quality research is needed to clarify its prognostic value across different patient subgroups and timeframes.

**Supplementary Information:**

The online version contains supplementary material available at 10.1186/s13054-025-05729-5.

## Background

 Weaning from mechanical ventilation is a major concern in Intensive Care Units (ICU). This process covers several steps [1]: spontaneous breathing trial (SBT), assessment of risk factors for extubation failure, cuff leak test and finally extubation. Considering the whole weaning process, from the SBT to the extubation, weaning can be defined as simple (successful extubation after one successful SBT), difficult (up to 3 SBT, or 7 days between first SBT and extubation), or prolonged (more than 3 SBT, or extubation more than 7 days after first SBT) [2].

Despite assessments for readiness and scheduling the extubation, from 15% [3, 4] to 30% [5, 6] of patients fail extubation. In this population of patients who fail the extubation, outcomes are very poor, with a high mortality [6]. Extubation failure can be defined as the need for reintubation from 24 h [7] up to 7 days [8, 9] following the extubation, when supported with non-invasive ventilation. Extubation failure can result from a variety of causes [10], including upper airway obstruction, congestive heart failure, respiratory failure, aspiration, and ineffective cough… This multifactorial nature makes it difficult to establish a clear underlying pathophysiology. Extubation failure can be classified as a “non-airway failure” or an “airway failure” [11]. Non-airway failure is related to the liberation from mechanical ventilation and is mostly represented by respiratory failure or congestive heart failure [11, 12]. Airway failure is related to the removal of the endotracheal tube and caused by an upper airway obstruction or an ineffective management of the upper airway or pulmonary secretions [11, 12]. About half of extubation failures would be related to an airway failure, and the other half with non-airway failure or mixed failure [12]. In both conditions, duration of mechanical ventilation, weak cough strength, and copious secretions were found to be associated with extubation failure [12]. These findings were also reported in a multivariate meta-analysis on risk factors of extubation failure [13], but which found that all these outcomes were related to a late extubation failure, between 48 h and 7 days post-extubation.

Just as the causes of extubation failure are multiple, the risk factors for these failures are also numerous, such as older age, impaired consciousness, positive fluid balance, abundant endotracheal secretions [10]. ICU Acquired Weakness (ICUAW) has been identified as one of these possible risk factors [14]. ICUAW is a muscle weakness described as “a secondary disorder while patients are treated for other life-threatening conditions” [15]. This affection would concern 33% of ICU patients [16]. The diagnosis of ICUAW is primarily clinical, with the use of a volitional manual muscle testing score involving limb muscles: the 60 points Medical Research Council-Sum Score (MRC-SS) [16]. Clinical diagnosis is made for a score below 48, and ICUAW is classified as severe for a score below 36 [17]. It was found that MRC-SS-defined ICUAW was associated with a longer duration of mechanical ventilation [18, 19], and also with extubation failure [20, 21]. A prospective observational study [22] highlighted that the group of patients with extubation failure presented more limb weakness as well as a higher prevalence of weak cough. However, association between limb muscles and respiratory muscles strength appears to be moderate to weak [20, 23–25]. Diaphragm dysfunction has been shown to occur independently of limb weakness [26]. Although peripheral muscle weakness has emerged as a potential factor to the weaning process, its precise role has not yet been clearly defined.

Apart from MRC-SS, another diagnosis tool for ICUAW is maximal handgrip strength (HGS). Best HGS cut-off values to diagnose ICUAW would be 11 kg for men and 7 kg for women, with a sensitivity of 80% and a specificity of 83% [27]. Still, HGS and MRC are moderately correlated, with reported coefficients of 0.5 [28], 0.55 [29] and 0.65 [27]. HGS would have a better inter-rater reliability than MRC-SS [30, 31], especially concerning highest level of this scale [27, 30, 31].

The first aim of the study was to investigate the association between maximal HGS and extubation outcome. Secondary aims included examining the association between maximal HGS and SBT outcome, as well as weaning classifications. Our hypotheses were that a low HGS was associated with poorer weaning outcomes, through a higher incidence of extubation failure, SBT failure, and difficult or prolonged weaning.

## Methods

This systematic review is based on the 2020 PRISMA guidelines [32] and the Checklist for critical Appraisal and data extraction for systematic Reviews of prediction modeling Studies (CHARMS) [33]. It was registered on Prospero with the number CRD42023471055.

Eligibility of the studies for this review was constructed according to a PICO strategy adapted for prognosis study: Population, Index of prognosis, Comparison, Outcome. The population of interest was adult patients hospitalized in an ICU who required mechanical ventilation for at least 24 h. The index of prognosis was the maximal HGS of the dominant hand before extubation. The primary outcome was the extubation failure or success. Secondary outcomes were SBT failure or success, and weaning type (simple, difficult or prolonged). A comparison against another index of prognosis was out of the scope of this study. Only cohort studies of prognosis were considered for inclusion. Randomized controlled trials or any other efficacy study were excluded.

The MedLine, EMBase, CINAHL and Cochrane Library databases were explored in December 2023, and updated in December 2024. The following Mesh terms were used: “hand strength” and “airway extubation”. Synonyms and boolean operators have also been used, depending on the database. No time limit or filter of was used.

Selection of the studies was conducted by two blinded reviewers (HDN, TG) within the help of the free version of rayyan.ai tool. Any disagreement was resolved by discussion, and remaining disagreements were resolved by consensus with consultation of a third reviewer (ND). According to the PRISMA flow diagram, the first step was checking and removing duplicates, followed by a first selection based on title and abstract, and a final selection based on full-text articles. Causes for the removal of full-text were reported.

Data extraction was released according to the CHARMS checklist [33] regarding the description of participants, outcomes to be predicted, prognosis factors, sample size, missing data, analysis, and results. Risk of bias was assessed by two blinded reviewers (HDN, TG), using the Quality In Prognosis Study (QUIPS) tool [34]. Discrepancies were addressed by the third reviewer (ND).

In the qualitative analysis, all studies were included, regardless of their risk of bias. The synthesis was built to report the mean differences of handgrip strength (i) between extubation success and extubation failure, (ii) between SBT success and SBT failure, and (iii) between different type of weaning groups (simple, difficult and prolonged). Results of receiver operating characteristics (ROC) analysis conducted to identify the best HGS threshold to predict weaning outcome were reported.

In the meta-analysis, studies with an overall high risk of bias as determined by the QUIPS were excluded. In order to pool mean differences of the studies, data presented as median and interquartile range were transformed into mean and standard deviation to pool the mean differences of the studies. This was done based on the hypothesis that data were normally distributed, and according to the third method described by Wan et al. [35]: the mean was estimated as (Q1 + Q2 + Q3)/3 and the standard deviation as IQR/1.35. Data from difficult and prolonged weaning groups were also pooled through a weighted average to create a “non-simple” weaning group [36].

Statistical analysis was conducted using 4.3.3 version of the R software, and the “metaphor” and “mada” packages. Heterogeneity was investigated through Cochrane’s Q test and heterogeneity index I^2^. When I^2^ was higher than 30%, a random effect model was used instead of a fixed effect model. The method used to pool the mean differences was inverse-variance in the fixed-effect model and Der Simonian and Laird for the random-effect model [37]. A sensitivity analysis was conducted through a leave-one-out process. The publication bias was assessed based on graphical representation of a funnel plot.

To assess the diagnostic accuracy of an HGS cut-off for predicting either extubation failure or non-simple weaning, we combined both outcomes into a single composite endpoint. A hierarchical summary ROC model, as described by Rutter and Gatsonis, was used for this pooled analysis [38]. Sensitivity and specificity extracted from the studies were standardized so that a positive test result corresponded to either extubation failure or non-simple weaning. As a sensitivity analysis, we then stratified the data and generated separate summary ROC curves for each outcome individually (one for extubation failure and one for non-simple weaning) to explore potential differences in diagnostic performance.

Certainty of evidence was estimated for each outcome using the Grading of Recommendations Assessment, Development and Evaluation (GRADE) approach adapted for prognosis factors [39].

## Results

### Search strategy

**Fig. 1:**
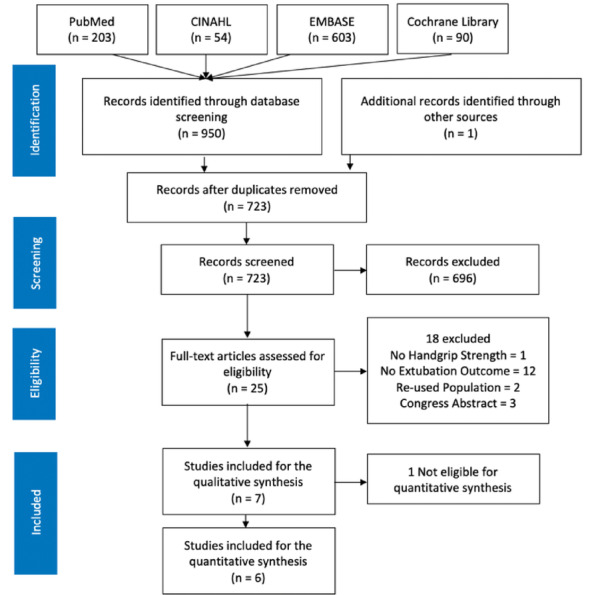
Flow diagram

From the database investigation, 950 records were found in December 2023. Upon actualizing the search strategy in December 2024, another article was found to meet the inclusion criteria and was added to the selection. After duplicates removal, 723 records were screened based on title and abstract, 25 articles were assessed for eligibility through the full-text, and 7 were included in the review. Reasons for exclusion were reported and presented in a flow diagram (Fig. 1).

### Risk of bias

**Fig. 2:**
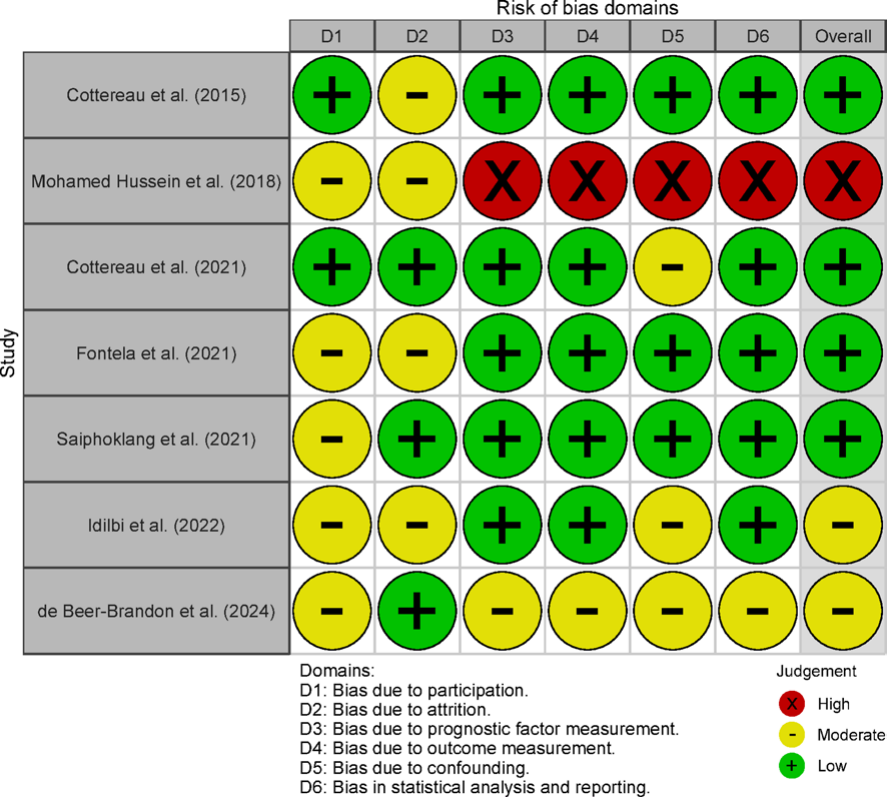
Risk of Bias

Raters agreed on 78% of the QUIPS tool. After reconciliation, four studies were considered with a low risk of bias, two with a moderate risk of bias, and one with high a risk of bias. Details for each domain of the QUIPS are presented in a traffic light plot (Fig. 2**)**.

### Characteristics of the studies

Among the seven studies included, the total number of patients was 707. Population characteristics are reported in Table 1.

**Table 1:** Study participants characteristics

Article	Country	Sample size	Sex (W/M)	Age	Gravity score	Reason for ventilation		DMV	Right handed	
Cottereau 2015	France	84	1.15	66 [53–79]	SAPSII 49 [37–63]	ARF		9 [4–17]	84%	
Mohamed Hussein 2018	Egypt	34	0.89	61 ± 12	NA	NA		9.7 ± 8.5	NA	
Cottereau 2021	France	233	0.68	66 [53–75]	SAPS II 53 [40–67]	ARF		6 [3–10]	90%	
Fontela 2021	Brazil	102	1.22	58 ± 18	APACHE II 24.8 ± 8.7	Pneumonia		1.5 [6–17]	86%	
Saiphoklang 2021	Thailand	93	1.38	72 ± 15	APACHE II 13.5±−4.7	Pneumonia		NA	94%	
Idilbi 2022	Israel	104	1.52	61 ± 19	NA	Metabolic		3.5 ± 3.3	NA	
De Beer-Brandon 2024	South Africa	57	0.68	45 ± 16	NA	NA		6.5+−4.4	NA	

Data are presented as Median [Interquartile Range] or Mean ± Standard Deviation

APACHE: Acute Physiology And Chronic Health Evaluation

ARF: Acute Respiratory Failure

DMV: Duration of Mechanical Ventilation

M: Men

NA: Not Applicable

SAPS: Simplified Acute Physiology Score

W: Women

HGS was measured by Jamar hydraulic hand dynamometer (Fred Sammons, Bolingbrook, Illinois) in five studies [40–44], and E.clear dynamometer EH101 (Camry, Hong Kong, China) in one study [36], and the tool used was not reported in one study [45]. The moments for HGS measurement were quite similar, mostly just before the first spontaneous breathing trial.

Extubation failure was defined as the need for reintubation for three studies [36, 41, 44] and extended to the need for unplanned non-invasive ventilation for three other studies [40, 42, 43]. One study did not give any precise definition [45]. Timeframes for considering extubation failure varied, ranging from 48 h in three studies [36, 40, 43, 45], up to 3 days in two studies [44, 45], and up to 7 days in one study [42]. Four studies [36, 40, 42, 43] used the categories from the international consensus conference on intensive care medicine from 2007 [2] separating simple weaning, difficult weaning and prolonged weaning.

Extubation failure occurred in a range from 6% up to 39%. HGS was statistically different between extubation failure vs. success in 2 of 6 studies (Table 2), and between simple vs. non-simple weaning in 3 of 4 studies (See Additionnal File 1).

**Table 2:** Maximal handgrip strength (kg) according to weaning outcomes

Article	Extubation		
	Failure	Success	*p*
Cottereau 2015	10 [5–18] *n* = 15 (18)	16 [7–23] *n* = 69 (82)	0.14
Mohamed Hussein 2018	2.8 ± 2 *n* = 7 (21)	17.3 ± 13.9 *n* = 27 (79)	**0.029**
Cottereau 2021	12 [8–20] *n* = 51 (22)	12 [6–20] *n* = 176 (78)	0.085
Saiphoklang 2021	8.3 ± 5.3 *n* = 6 (6)	16.3 ± 6.5 *n* = 87 (94)	**0.004**
Idilbi 2022	18.5 ± 11.7 *n* = 14 (14)	28.0 ± 14.2 *n* = 89 (86)	NA
De Beer-Brandon 2024	7.69 ± 6.84 *n* = 22 (39)	13.31 ± 12.13 *n* = 35 (61)	0.052

Data are presented as Median [Interquartile Range] or Mean ± Standard Deviation. **Bold**: p<0.05

NA: Not Applicable

## Meta-analysis

### Difference of handgrip strength between extubation failure vs. success

**Fig. 3:**
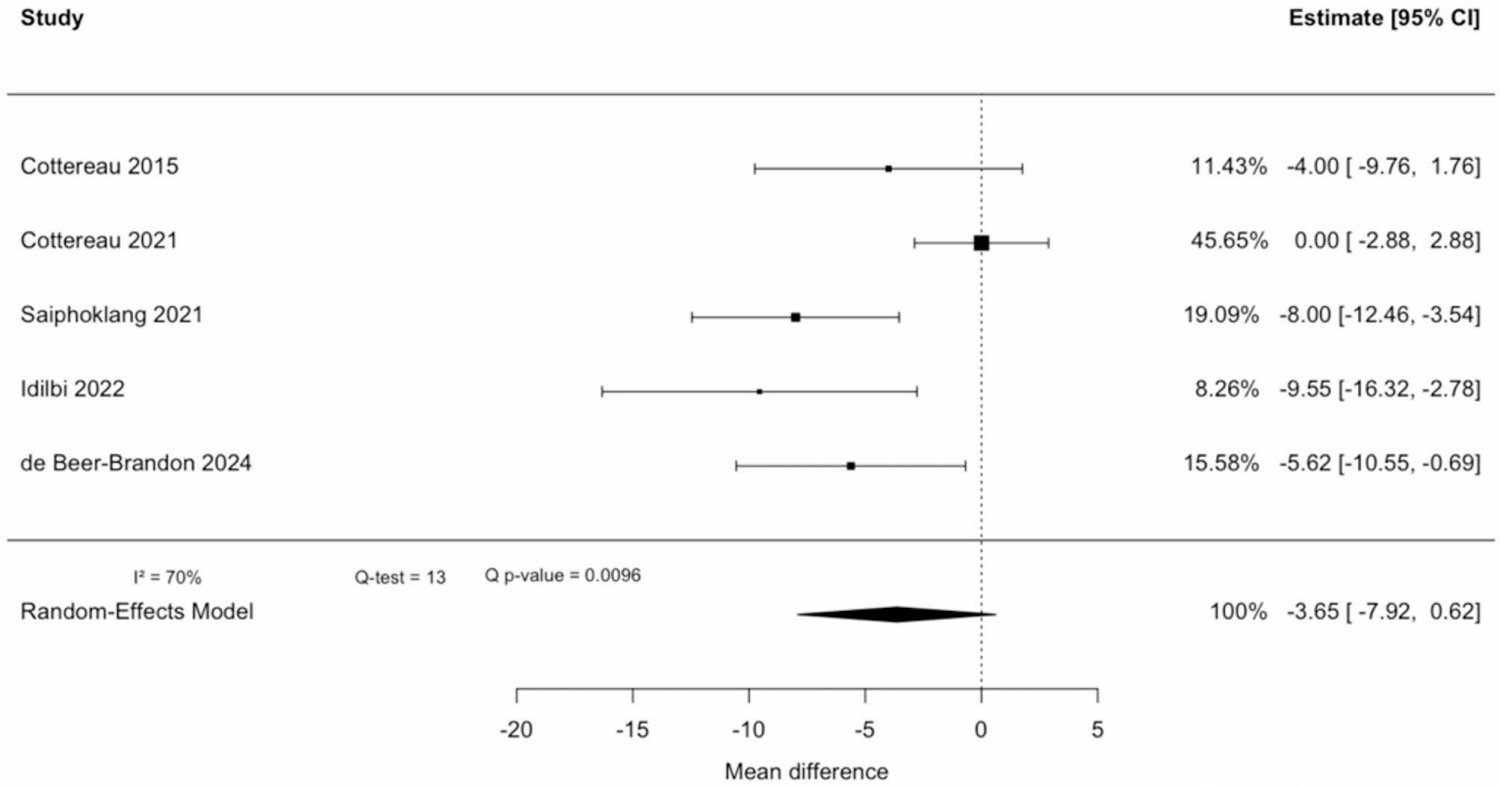
Handgrip strength pooled mean difference between extubation failure and success

The five considered studies represented 549 patients. Heterogeneity was found to be high (I^2^ = 70%) and a random-effect model was used. The pooled estimated mean difference in maximal HGS prior to the SBT between patients with extubation failure and those with extubation success was − 3.62kg (95% CI: −7.92 to 0.62), indicating no statistically significant difference, as the confidence interval includes zero (Fig. 3). The weight of the Cottereau et al.’ study [42] was the most important in the model (46%). Sensitivity analysis and funnel plot (see Additional file 2) revealed that this article generated most of the heterogeneity in the meta-analysis, and could have been considered as an outlier.

The quality of evidence is low to very low for the absence of a significant difference in HGS between patients with extubation failure or success, owing to risk of bias from unadjusted results, study inconsistency, and potential publication bias.

### Difference of handgrip strength for SBT failure vs. success

**Fig. 4:**
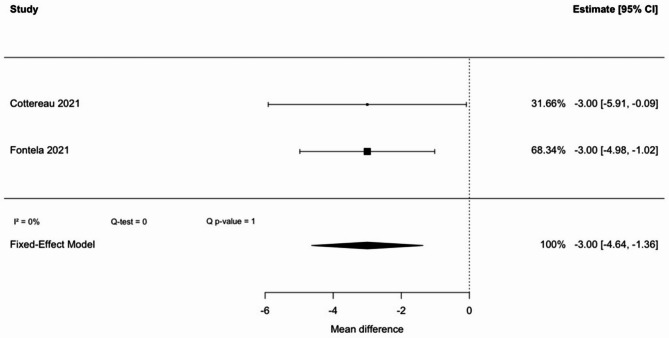
Handgrip strength pooled mean difference between SBT failure and success

Comparison of HGS between patients who succeeded in the SBT and those who failed included two studies, with a total of 313 patients (Fig. 4). This result was statistically significant, with a low heterogeneity (I^2^ = 0%). Pooled mean difference was − 3.00 kg (95% CI: −4.64 to −1.36), indicating that weak HGS is associated with an increased rate of SBT failure.

Owing to risk of bias from unadjusted results, limited studies, and indirect evidence, the quality of this evidence is low.

### Difference of handgrip strength for non-simple vs. simple weaning

**Fig. 5:**
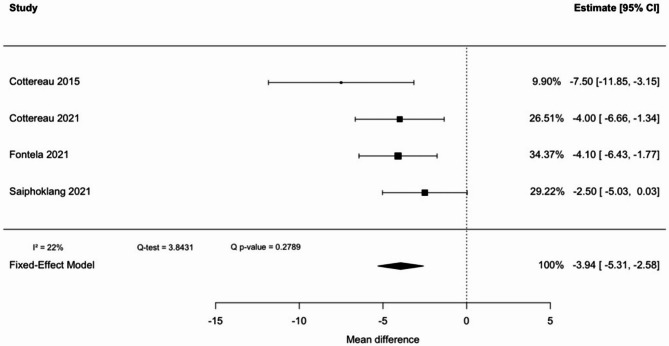
Handgrip strength pooled mean difference between non-simple and simple weaning

To compare non-simple weaning group versus simple weaning group, four studies were considered, with a total of 490 patients (Fig. 5). There was a statistically significant difference, with a low heterogeneity (I^2^ = 22%). There was a pooled mean difference of −3.94 kg (95% CI: −5.31 to −2.58) in HGS, thus showing that a lower HGS favors a non-simple weaning.

Despite the risk of bias from unadjusted results, there is moderate-quality evidence supporting that HGS is lower in individuals with non-simple weaning compared to those with simple weaning.

### Diagnosis accuracy of HGS-defined ICUAW to predict extubation failure or non-simple weaning

Abilities of HGS to predict extubation failure or non-simple weaning through prognosis validity was reported in 5 studies. Extubation failure as an endpoint was considered by three studies [42–44] and non-simple weaning in two others [36, 40], representing a sample of 594 patients. The cut-off for diagnosing ICUAW was predetermined as 11 kg [40, 42, 44] or 14 kg [36] for men, and 7 kg [40, 44] or 8 kg for women [36, 42]. One study [43] used a ROC curve to determine the best cut-off for extubation failure prediction which was 12.7 kg with a sensitivity of 76% and a specificity of 83.3% [40, 42, 44]. 

**Fig. 6:**
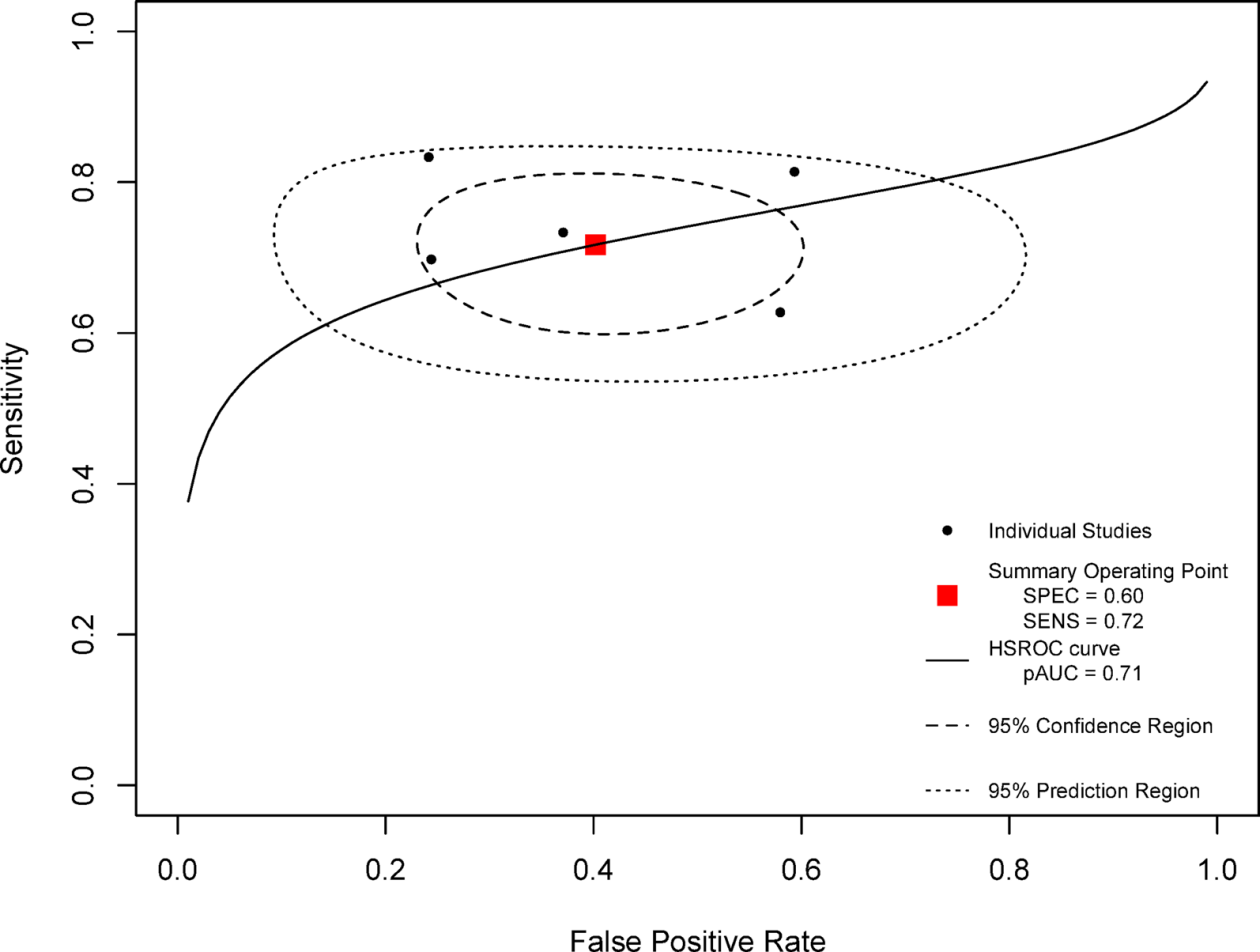
HSROC Curve of handgrip strength to predict non-simple weaning or extubation failure. HSROC = Hierarchical Summary Receiver Operating Characteristic, SPEC = specificity, SENS = sensitivity, pAUC = partial Area Under the Curve

The summary ROC curve partial area under the curve (AUC) was 0.71 (Fig. 6), thus showing a good ability to predict the outcome. Overall sensitivity was 0.72 and specificity 0.60. The sensitivity analysis comparing extubation failure or non-simple weaning as different endpoints found similar characteristics (see Additional file 3). Respectively for the predictions of extubation failure and non-simple weaning, partial AUC were 0.73 and 0.75, sensitivities were 0.73 and 0.76, and specificities were 0.61 and 0.59.

Assuming a pre-test probability of extubation failure of 10%, positive and negative predictive values were respectively 17% and 95%, and 31% and 90% when the pre-test probability was 20%.

## Discussion

### Major findings

To the best of our knowledge, this is the first systematic review and meta-analysis evaluating maximal HGS as a predictor of weaning outcomes in ICU patients. No statistically significant difference in HGS was observed between patients who succeeded or failed extubation (pooled mean difference: − 3.62 kg; 95% CI: − 7.92 to 0.62). While the trend suggests lower HGS in patients with extubation failure, the confidence interval includes zero, and the finding must be interpreted as negative. Significant differences were found in secondary analysis regarding SBT outcome and weaning classification, with pooled mean differences of − 3.94 kg (95% CI: − 5.31 to − 2.58) and − 3.00 kg (95% CI: − 4.64 to − 1.36), respectively.

Our primary analysis did not find an association between HGS weakness and extubation failure, which contrasts with our initial hypothesis. This hypothesis was supported by prior studies demonstrated associations between extubation failure, and ICUAW assessed by the MRC-SS [20]. Extubation failure is a multifactorial event, which can be caused by factors unrelated to peripheral muscle strength, such as upper airway obstruction, respiratory or cardiac dysfunction, excessive secretions, or impaired consciousness [10]. These factors may dominate the clinical picture and lead to failure regardless of a patient’s peripheral muscular status. For instance, a patient with preserved HGS but poor cough effectiveness or abundant secretions may still fail extubation due to inadequate airway protection or clearance.

Sensitivity analysis excluding the only study [42] that used a 7-day definition of extubation failure revealed that HGS weakness was then significantly associated with extubation failure. This suggests that HGS may help predict early extubation failure, though its predictive value may decrease over time, consistent with previous evidence [13] which showed that risk factors for extubation failure differ depending on the timing considered. The 7-day time frame in that study was chosen because prophylactic NIV was delivered to patients deemed at high risk of extubation failure. Weak patients who received this additional support may have avoided extubation failure, although the actual benefit of prophylactic NIV in this subgroup remains uncertain. Furthermore, in this study, the use of curative NIV was classified as extubation failure, and 12 patients in this group did not require reintubation. This may inflate the reported extubation failure rate, and blur the clinical distinction between patients who truly failed extubation and those who were successfully managed with additional ventilatory support. However, patients requiring post-extubation curative NIV are more likely to exhibit muscle weakness [46]. Therefore, although the extubation failure rate may be overestimated, the average HGS within this group is unlikely to have been significantly overestimated.

The diagnostic accuracy of HGS in predicting extubation failure or non-simple weaning showed a sensitivity of 72% and a specificity of 60%, with positive predictive values ranging from 17% to 31%, and negative predictive values from 90% to 95%. The thresholds proposed by Ali et al. [27]—11 kg for men and 8 kg for women—provide practical reference points to identify patients at risk. Although a positive test result (presence of weakness) has limited predictive value, the high negative predictive value supports the usefulness of HGS in ruling out extubation failure, thereby reinforcing clinical decisions when HGS is preserved. However, physicians and ICU healthcare providers tend to be more accurate in identifying patients who are likely to succeed with extubation rather than those who are at risk of failure [22].

All these considerations may reflect the limited physiological overlap between peripheral and respiratory muscle function. Furthermore, we propose that the discrepancy between our findings and those based on MRC-SS may be explained by the distribution of muscle weakness. Distal weakness, such as reduced grip strength, may have less impact on weaning success compared to proximal weakness, which might be more directly linked to impaired trunk control, respiratory muscle function, and swallowing. This hypothesis is supported by recent findings. MRC-diagnosed ICUAW was found to be associated with an adjusted relative risk of 11.2 for post-extubation dysphagia [47], and with odds ratios of 5 to 9 for post-extubation aspiration and pharyngeal dysfunction [48]. In the latter study, MRC-SS was moderately correlated with HGS (*r* = 0.5), but HGS alone could not predict aspiration risk.

While current evidences do not justify the clinical use of HGS alone to guide extubation decisions, it may still provide complementary information when integrated into multifactorial weaning assessments. Moreover, HGS measurement remains a simple, quick, and reliable tool at the bedside [30, 31]. It can even be performed using low-cost, low-tech devices [49], making it an attractive option in various ICU settings.

Building on these findings, future high-quality prospective studies should assess the predictive performance of HGS in relation to weaning outcomes, using appropriate statistical models within multivariable prognostic frameworks. These models should integrate key risk factors for weaning failure, including neurological status, respiratory function, cardiac function, upper airway obstruction, diaphragm dysfunction, cough strength, amount of secretion, and dysphagia. Establishing clinically relevant thresholds and integrating HGS into composite prediction tools could enhance both the accuracy and the bedside applicability of weaning assessments.

### Limitations

The external validity of this finding may be limited to a specific subset of ICU patients. Indeed, performing HGS measurement requires patients to be sufficiently conscious and cooperative to follow instructions—criteria that may introduce a selection bias. Yet, impaired consciousness is a well-established risk factor for extubation failure [13]. Moreover, patients with pre-existing neuromuscular, rheumatologic, or orthopedic conditions were excluded from the included studies, likely omitting individuals with multiple comorbidities, particularly neuromuscular disorders, who are known to be at higher risk of weaning failure [50].

In the ICU setting, the standard error of HGS measurement has been reported to range from 2.8 kg to 4.5 kg [31]. The differences observed in our study fall within this range, which may limit the ability to accurately classify patients at the bedside.

The number of studies included in this meta-analysis was limited. Although meta-analytic pooling was feasible, the stability of the results may change as more studies become available [51]. Statistical heterogeneity was observed across studies, likely driven by clinical variability, particularly differences in the definition of extubation failure [52].

It would have been pertinent to consider the reintubation as an endpoint in the sensitivity analysis, given its clinical relevance and association with poor patient outcomes [10]. It was not possible because only two studies reported HGS according to reintubation status. One of these studies was assessed at very high risk of bias [41]. In the other study [44], HGS was significantly lower in patients who required reintubation within 3 days post-extubation, but this association did not remain in the multivariate analysis.

While three studies reported and accounted for potential confounders in multivariate analyses [36, 40, 45], adjusted estimates could not be incorporated into this meta-analysis, limiting causal inference.

Finally, a formal test for small-study effects could not be performed due to the small number of included studies. However, visual inspection of the funnel plot suggested potential publication bias, possibly due to a lack of small studies reporting negative findings. Alternatively, the asymmetry may have been driven by the disproportionate influence of a single large study [53].

## Conclusion

This systematic review and meta-analysis suggests that maximal HGS is not significantly associated with extubation failure (mean difference: − 3.62 kg; 95% CI: − 7.92 to 0.62).

Given the low certainty of evidence and heterogeneity across studies, further high-quality research is needed to clarify the prognostic role of HGS, particularly in relation to the timing of extubation failure and the influence of proximal versus distal muscle weakness.

While this evidence does not justify the clinical use of HGS alone to guide extubation decisions, it may still provide complementary information when integrated into multifactorial weaning assessments.

## Supplementary Information

Supplementary Material 1

Supplementary Material 2

Supplementary Material 3

## Data Availability

The datasets used and/or analysed during the current study are available from the corresponding author on reasonable request. This review was only based on already available data from the included articles and their supplementary materials.

## Abbreviations

AUC: Area under the curve
CHARMS: Checklist for critical appraisal and data extraction for systematic reviews of prediction modeling studies
GRADE: Grading of recommendations assessment, development and evaluation
HGS: Hand grip strength
ICU: Intensive are unit
ICUAW: Intensive care unit-acquired weakness
MRC-SS: Medical research council - sum score
PICO: Population index of prognosis comparison outcome
PRISMA: Preferred reported items in systematic reviews and meta-analyses
QUIPS: Quality in prognosis study
ROC: Receiver operating characteristics
SBT: Spontaneous breathing trial
