# Supplementary material for: Handgrip strength association with weaning outcome in mechanically ventilated ICU patients: a systematic review and meta-analysis
Source: Crit Care. 2025 Nov 7;29:478. doi: 10.1186/s13054-025-05729-5 (PMC12598822; doi:10.1186/s13054-025-05729-5)
Supplement: Supplementary file 1 — Supplementary Material 1 [file 13054_2025_5729_MOESM1_ESM.docx]

| Maximal Handgrip Strength (kg) according to weaning outcomes | | | | | | | | | | | | | |
| --- | --- | --- | --- | --- | --- | --- | --- | --- | --- | --- | --- | --- | --- |
| Article |  | Extubation | | |  | First SBT | | |  | Weaning | | | |
|  |  | Failure | Success | *p* |  | Failure | Success | *p* |  | Simple | Difficult | Prolonged | *p* |
| Cottereau 2015 |  | 10 [5-18]  n=15 (18) | 16 [7-23]  n=69 (82) | .14 |  | - | - |  |  | 20 [12-26]  n=41 | 12 [6-21]  n=33 | 6 [3-11]  n=10 | **.008** |
| Mohamed Hussein 2018 |  | 2.8±2  n=7 (21) | 17.3±13.9  n=27 (79) | **.029** |  | - | - |  |  | - | - | - | - |
| Cottereau 2021 |  | 12 [8-20]  n=51 (22) | 12 [6-20]  n=176 (78) | .085 |  | 8.5 [2.5-16]  n=60 | 12 [5-18]  n=171 | .057 |  | 12 [6-20]  n=164 | 8 [2-14]  n=67 | | **.0012** |
| Fontela 2021 |  | - | - | - |  | 4.9 [2.9-7.2]  n=30 | 6.9 [3.2-12.6]  n=72 | .060 |  | 7.5 [3.5-14.6]  n=59 | 4.9 [2.6-7.2]  n=43 | | **<.001** |
| Saiphoklang 2021 |  | 8.3±5.3  n=6 (6) | 16.3±6.5  n=87 (94) | **.004** |  | - | - | - |  | 16.36 ± 7.17  n=72 | 13.73 ± 4.69  n=19 | 15.1±0.42  n=2 | .318 |
| Idilbi 2022 |  | 18.5±11.7  n=14 (14) | 28.0±14.2  n=89 (86) | NA |  | - | - | - |  | - | - | - | - |
| De Beer-Brandon 2024 |  | 7.69±6.84  n=22 (39) | 13.31±12.13  n=35 (61) | .052 |  | - | - | - |  | - | - | - | - |
| Data are presented as Median [Interquartile Range] or Mean ± Standard Deviation  SBT: Spontaneous Breathing Trial  NA: Not Applicable | | | | | | | | | | | | | |

**Additional File 1: Maximal Handgrip Strength (kg) according to weaning outcomes**
