# Supplementary material for: Handgrip strength association with weaning outcome in mechanically ventilated ICU patients: a systematic review and meta-analysis
Source: Crit Care. 2025 Nov 7;29:478. doi: 10.1186/s13054-025-05729-5 (PMC12598822; doi:10.1186/s13054-025-05729-5)
Supplement: Supplementary file 2 — Supplementary Material 2 [file 13054_2025_5729_MOESM2_ESM.docx]

**Additional File 2**

**Sensitivity Analysis: Leave-one-out process**

Acronyms:

- EF: Extubation Failure
- ES: Extubation Success
- HGS: Hand Grip Strength
