# Supplementary material for: Handgrip strength association with weaning outcome in mechanically ventilated ICU patients: a systematic review and meta-analysis
Source: Crit Care. 2025 Nov 7;29:478. doi: 10.1186/s13054-025-05729-5 (PMC12598822; doi:10.1186/s13054-025-05729-5)
Supplement: Supplementary file 3 — Supplementary Material 3 [file 13054_2025_5729_MOESM3_ESM.docx]

**Additional File 3**

**Sensitivity Analysis of binary HGS diagnosis accuracy in predicting extubation failure and non-simple weaning**

Acronyms:

HSROC= Hierarchical Summary Receiver Operating Characteristic, SPEC=specificity, SENS=sensitivity, pAUC=partial Area Under the Curve
